# Latent classes of early response trajectories to biologics initiation in juvenile idiopathic arthritis: an analysis of four trials

**DOI:** 10.1186/s12969-022-00719-1

**Published:** 2022-07-30

**Authors:** Lily Siok Hoon Lim, Shamsia Shobhan, Armend Lokku, Sarah Ringold, Eleanor Pullenayegum

**Affiliations:** 1 Department of Pediatrics, Children’s Hospital Research Institute of Manitoba, University of Manitoba, 501F-715 McDermot Ave, Winnipeg, Manitoba R3E 0V9 Canada; 2 Department of Community Health Sciences, University of Manitoba, Winnipeg, Canada; 3 Dalla Lana School of Public Health, University of Toronto, Toronto, Canada; 4 Seattle Children’s Hospital, Seattle, USA; 5 Child Health Evaluative Sciences, SickKids, Toronto, Canada

**Keywords:** Juvenile idiopathic arthritis, Polyarticular-course, Biologics, Treatment, Response, Disease activity trajectory, Trials

## Abstract

**Aims:**

1) To delineate latent classes of treatment response to biologics in juvenile idiopathic arthritis (JIA) patients in the first 16 weeks after initiation. 2) To identify predictors of early disease response.

**Methods:**

The study population was drawn from four biologics trials in polyarticular course JIA: Etanercept 2000, Abatacept 2008, TRial of Early Aggressive Therapy (TREAT) 2012 and Tocilizumab 2014. The outcome was active joint counts (AJC). Semiparametric latent class trajectory analysis was applied to identify latent classes of response to treatment; AJC was transformed for this modelling. We tested baseline disease and treatment characteristics for their abilities to predict class membership of response.

**Results:**

There were 480 participants, 74% females. At baseline, 26% were rheumatoid factor positive. 67% were on methotrexate at enrollment. Three latent class solution provided the best fit. Baseline AJC was the sole best predictor of class membership. Participants classified by their highest membership probabilities into high baseline AJC (> 30) and slow response (26.5%), low baseline AJC (< 10), early and sustained response (29.7%), and moderate baseline AJC progressive response (43.8%). Participants were classified into the latent classes with a mean class membership posterior probability of 0.97. Those on methotrexate at baseline were less likely to belong to high baseline AJC class.

**Conclusions:**

Three latent classes of responses were detectable in the first 16 weeks of biologics therapy. Those with the highest baseline AJC demonstrated very slow response in this window and were less likely to be on concomitant methotrexate.

**Trials registration:**

TREAT 2012 (NCT NCT00443430) (Wallace et. al, Arthritis Rheum 64:2012–21, 2012), tocilizumab trial 2014 (NCT00988221), abatacept trial 2008 (NCT00095173). Etanercept 2000 from Amgen does not have a trial registration number.

**Supplementary Information:**

The online version contains supplementary material available at 10.1186/s12969-022-00719-1.

Juvenile idiopathic arthritis (JIA) is the most common childhood-onset chronic rheumatic disease [1]. In the USA alone, 300,000 children have been estimated to be living with JIA [1]. JIA is a heterogenous disease and latent disease activity trajectories and disease courses have been demonstrated among patients with JIA, even with the same disease subtype [2–4].

Biologics are used for treatment of JIA that is not fully responsive to first-line disease modifying anti-rheumatic drugs (DMARDs), usually methotrexate (MTX). Currently, the aim of JIA treatment is to achieve disease control, preferably remission (no disease activity) [5]. There is a concept of a window of opportunity in early arthritis treatment where the chance of long-term remission is higher if inactive disease can be attained early [6]. Therefore, if individuals who are unlikely to respond to a treatment are identified early, their treatment can be changed earlier, bringing their disease into remission as soon as possible, within the window of opportunity. We demonstrated recently among biologic DMARD (bDMARD) trial participants that if an individual had not responded with at least a pediatric American College of Rheumatology 50% (ACR50) response by 3 months, this individual has a very low probability to respond even if we waited longer [7].

As clinicians, we have observed heterogeneity in clinical responses to treatment. Heterogeneity in disease courses have been demonstrate in JIA as well as other diseases [2, 8–10]. Prior studies in JIA have demonstrated latent classes of disease courses. However, therapeutic response to specific classes of medications, especially early following treatment initiation, have not been studied in previous JIA studies using latent class methods [8, 11]. There might be unobserved (latent) patterns of response following bDMARD treatment, where groups of patients demonstrating similar patterns of response may cluster.

In this study, we will examine whether there are latent patterns of active joint counts (AJC) trajectories in the first 16 weeks following initiation of a bDMARD among trial participants with polyarticular course JIA. We will test for predictors of AJC response classes.

## Methods

### Population & Study Design

The study population was drawn from four JIA trials: Etanercept 2000 (Amgen) [12], Abatacept (Bristol-Meyers Schwab, BMS) 2008 [13], Tocilizumab (Roche) 2014 [14], TRial of Early Aggressive Therapy (TREAT, Childhood Arthritis Rheumatology Research Alliance CARRA) 2012 [15]. All four trials recruited JIA patients with polyarticular course disease. TREAT and Tocilizumab 2014 recruited from 2 to 17 years old while Etanercept 2000 and Abatacept 2008 recruited from 6 to 17 years old. Polyarticular course JIA patients included those with rheumatoid factor (RF) positive or negative polyarthritis, extended onset oligoarticular JIA and systemic JIA without systemic features. Enthesitis-related-arthritis patients and juvenile psoriatic arthritis patients were excluded for all the trials except for TREAT. The TREAT trial allowed children without psoriasis, with a positive family history of psoriasis to be included [15]. All were multi-centre, international trials, except for TREAT which was limited to USA centres. Except for TREAT, which used a parallel-arm trial design, the other three trials gave the test biologics to all participants in the first 16 weeks (12 weeks for Etanercept 2000). For the TREAT trial, we used only the participants in the arm that received Etanercept, which was given with prednisone and MTX [15]. Additional details about study designs of each trial summarized in Appendix Table A1. Deidentified trial data were provided by CARRA, Amgen and BMS. Data from these three trials were then combined with the Hoffman-La Roche (Tocilizumab) trial data which were housed on Vivli, a secure global clinical research data sharing platform [16]. Data analysis was performed on Vivli. This study was approved by the Bannatyne campus research ethics board at the University of Manitoba (HS20486).

We used a longitudinal cohort design for this study, studying response in the first 16 weeks after starting a biologic. Participants were assessed at 2- to 4-weekly intervals in the first 16 weeks.

### Primary outcome & covariates

The primary outcome was AJC, measured at every visit. For covariates, we tested clinical characteristics at baseline including age at diagnosis, duration of disease, baseline AJC, erythrocyte sedimentation rate (ESR), rheumatoid factor status, MTX and prednisone treatment. This is a shorter list of covariates then the usual amount considered in observational cohorts as the trials only collected a limited, pertinent dataset. Also, these were the variables available in common from all four of the trial datasets.

### Statistical methods

Summary statistics for continuous variables in mean (standard deviation, SD) or median (25th- 75th percentile) were presented as appropriate. Proportions were reported for categorical variables. Comparisons were made using chi-square test for categorical variables and Kruskal-Wallis test for continuous variables.

The AJC was transformed to log (AJC + 1.8) to assume an approximately Normal distribution. We applied a growth mixture model, a parametric form of latent class trajectory analysis, to identify latent classes of response trajectories [17, 18]. The response trajectory is latent as it cannot be directly observed. The best fitting shape for the mean trajectory was a cubic model using a equidistance spline function with 10 nodes [19]. We started with two latent classes, then increased to six. We chose the best fitting number of latent classes guided by the nadir of Bayesian Information Criterion (BIC), smallest group not < 10% of the total population and by clinical experience [20, 21]. Single covariates were incorporated into the growth mixture model as a membership predictor. The covariate resulting in the smallest BIC was retained as the base membership predictor, then covariates were added on iteratively. Additional covariates would be kept if the resulting Akaike Information Criterion (AIC) improved over the prior membership predictor(s) [22]. Every individual has a probability of belonging to each of the latent classes; probabilities for each individual add up to 1.0. For reporting latent class membership, we classified each individual into the latent class that they have the highest probability of belonging to. All analyses were conducted in R, using the lcmm package, on the Vivli platform [16, 23, 24].

## Results

### Population

Four hundred eighty children were included: 68 from Etanercept 2000, 42 from TREAT, 180 from Tocilizumab 2014 and Abatacept 190. See Table 1 for baseline demographics and clinical characteristics of the combined study population. There were 2662 visits. Majority (469/80, 97%) had ≥4 visits.

**Table 1:** Baseline demographics and clinical characteristics of study population

Character	Results
Females (%)	353 (74)
Median age in years at diagnosis (25-75th percentile)	6.32 (3.05–10.00)
Median years in disease duration (25-75th percentile)	4.04 (2.00–8.00)
Median baseline AJC (25-75th percentile)	16.00 (9.00–27.00)
Median baseline ESR (25-75th percentile)	28 (13–47)
Baseline RF positive (%)	124 (26)
Baseline MTX treatment (%)	322 (67)
Baseline prednisone treatment (%)	214 (45)

### Latent AJC response trajectories

**Fig. 1:**
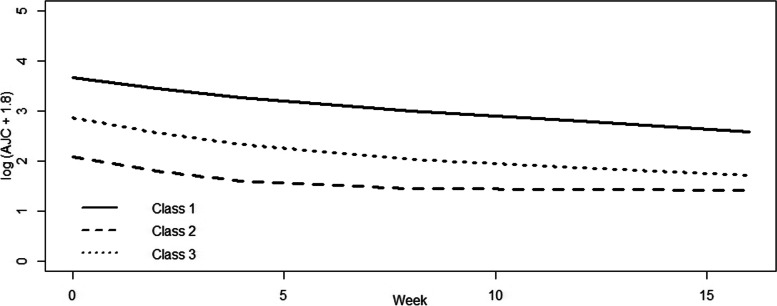
Early Latent Trajectories of Response in Juvenile Idiopathic Arthritis Patients Following Biologic DMARDs, Predicted by Baseline Active Joint Count. Legend: Class 1 (26.5%) was high baseline AJC with slow response. Class 2 (29.7%) was low baseline AJC with early fast response. Class 3 (43.8%) was moderate baseline AJC, with steady improvement

Three latent class trajectories was the best solution according to our pre-defined criteria (Appendix Fig. A1). When univariable membership covariates were tested within the latent class model, the baseline AJC was the best performing covariate (Appendix Table A2). When we added covariates (tested two and three membership covariates combinations) to the baseline AJC model, none performed better than the baseline AJC model. Therefore, we presented latent class trajectories probabilities as predicted only by baseline AJC (Fig. 1). Study participants were classifiable into three latent classes: 26.5% were in class 1 where there was a high baseline AJC and slow response, 29.7% were in class 2 where there was a low baseline AJC and quick early response then sustained plateaued response, and 43.8% were in class 3 where there was a moderate level of baseline AJC, early and progressive response over time. The rates of response (slopes) between the low (class 2) and moderate (class 3) were different. The mean posterior probability of membership in class 1 and 2 were both 0.97 and in class 3 was 0.96 (Appendix Table A3). Ninety-five percent of patients in class 1, 94% in class 2 and class 3 had a posterior probability of membership of ≥0.80. Overall, 450 (n_2_, 94%) had a posterior probability of membership of ≥0.80. We included the latent class trajectory model results without a membership predictor in the Appendix (Fig. A1).

**Fig. 2:**
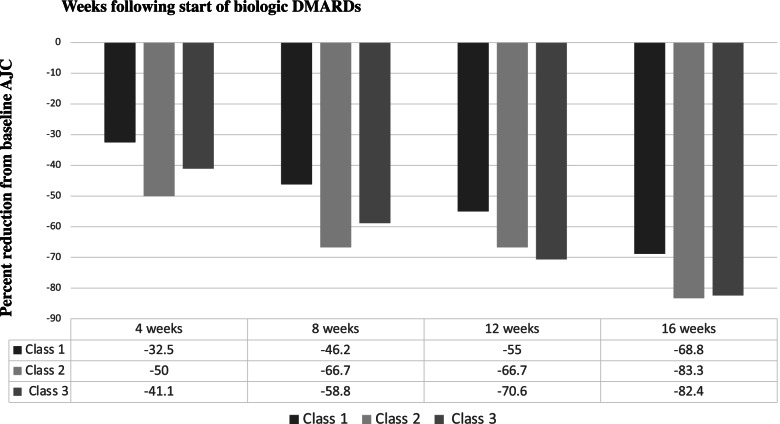
Percent Reduction in Median Active Joint Counts in the First 16 Weeks Following Biologic DMARDs, by latent classes (n_2_ = 450, probability≥0.80)

Figure 2 demonstrated the precent reduction in median AJC over time, in individuals by their assigned latent classes (using only n_2_, with highest membership probability ≥0.80). For class 1 patients, AJC reduced by 33% by week 4 but those in class 2 and 3 reduced by 50 and 41% respectively. By week 16, class 1 patients reduced their AJC by 69% from baseline levels but those in classes 2 and 3 have reduced by 83 and 82% respectively. The distribution of median AJC by latent classes (n_2_) over 16 weeks was presented in the Appendix (Fig. A2 and Table A4).

Table 2 showed the distribution of baseline clinical characteristics across the latent classes. The median baseline AJC of those assigned to class 1 was higher compared to those in assigned to class 2 (40 vs 6) or even class 3 (40 vs 17). Using the lower bounds of the 25th percentile of median baseline AJC as cut-offs, we can divide and envision patients’ potential latent response trajectory classes according to their baseline AJC of > 30 (high baseline AJC class 1), < 10 (low baseline AJC class 2) and 10 ≤ baseline AJC ≤ 30 (moderate baseline AJC class 3). The proportion of those in class 1 (high baseline AJC) on baseline MTX was lower than patients following the other two latent classes, which showed more rapid and substantial early response.

**Table 2:** Distribution of Baseline Clinical Characteristics in the Early Latent Response Classes of Juvenile Idiopathic Patients Following bDMARDs^a^

	Class 1 High baseline AJC, slow response *n* = 120	Class 2 Low baseline AJC, early and sustained response *n* = 133	Class 3 Moderate baseline AJC, early and progressive response *n* = 197	Differences between classes P^**b**^
**Median disease duration in years 25th–75th percentile**	4.0 (2.0–8.0)	3.7 (2.0–6.9)	4.0 (2.0–8.0)	0.135
**Median baseline AJC 25th–75th percentile**	40 (32–47)	6 (5–8)	17 (14–20)	< 0.001
**Median baseline ESR 25th–75th percentile**	38 (23–58)	19 (10–39)	28 (13–45)	< 0.001
**Number (%) positive rheumatoid factor**	30 (25.0)	31 (23.3)	58 (29.4)	0.003
**Number (%) on methotrexate**	62 (51.7)	106 (79.7)	131 (66.5)	< 0.001
**Number (%) on prednisone**	52 (43.3)	52 (39.1)	94 (47.7)	0.002

AJC, active joint counts. ESR, erythrocyte sedimentation rate

^a^ Only those with a posterior probability of membership in a class ≥0.80 presented in this table

450 (out of 480) participants were classifiable by this rule

The p column represented an overall study population-level comparison, this does not imply significance as a membership predictor between groups. For significance of the above as latent class membership predictors, please refer to Table A2 (Appendix)

^b^ Continuous variables were compared using Kruskal-Wallis test and categorical variables with chi-square test

With class 3 (moderate baseline AJC) as the reference class, higher AJC was associated with higher odds of being in class 1 (OR 1.92, 95% CI 1.26–2.93) and lower odds of being in class 2 (OR 0.07, 95% CI 0.00–0.93) (Appendix Table A2).

## Discussion

In this study using bDMARD trial data from polyarticular course JIA patients, we found three latent classes of early response following the start of a bDMARD. Baseline AJC was the best predictor of membership in the three latent classes. Those with high baseline AJC had a slow response throughout the first 16 weeks. Those with moderate and low baseline AJC had fast early response ~ 4 weeks, Appendix Fig. A2). While those with moderate baseline AJC continued to respond steadily, albeit at a slower rate for the rest of the time (5–16 weeks), those in the low baseline AJC group plateaued but sustained their response following their initial response during this early observation period.

Our study is unique as we used closely collected (2- to 4-weekly) early response data from JIA bDMARD trials in the first 12–16 weeks following the initiation of bDMARD. This approach allows the study of the anatomy of early treatment response for the first time. This is also the first time that latent trajectories of response to specific bDMARD treatment are identified. While disease activity trajectories have been identified in JIA in the past, most were performed on retrospective cohorts except for one Canadian cohort [8, 25, 26]. Their purpose was to describe patients’ disease courses. The periods of observation were much longer (up to 10 years). Treatment effects were implicit in the derived latent trajectory classes and not targeted to specific drugs. In contrast, we used the latent classes to characterize patients’ response following initiation of bDMARD, with an aim towards personalizing therapy, as informed by individuals’ clinical characteristics, to accelerate the time to improvement.

We found that those with very high baseline disease activity responded slowly during the first 16 weeks. In fact, by the end of the observation period, they still had a median AJC of 12.5, which represented 69% reduction in AJC from baseline but this was still a significant burden of disease activity, especially when compared to patients in the other two groups. With the discovery of this high AJC group, perhaps this group should be the target of a different approach to treatment. We noted that there was no difference in the proportion of patients being treated with prednisone at baseline in class 1 compared to class 3 (moderate baseline AJC), though more in class 1 received prednisone than those in class 2 (low baseline AJC). In univariable testing, baseline prednisone did not predict membership in one latent class compared to another (Table A2). Significantly fewer patients in class 1 were on MTX (52%) at the start of bDMARD compared to the other two groups (67–80%). In univariable analysis, being on MTX was associated with lower likelihood of belonging to class 1 (high baseline AJC) compared to class 3 (moderate baseline AJC, OR 0.22, 95% CI 0.12–0.41). However, being on MTX at baseline did not have an additional predictive effect on class membership beyond that of the baseline AJC. Taken together, barring practice variations in MTX use among pediatric rheumatologists around the world, perhaps this could be interpreted as that even if MTX as the lone DMARD is unable to control disease activity, those on MTX have a lower chance of belonging to a high disease activity class that does not respond quickly or well to bDMARD treatment. Some may argue that patients who were not treated with MTX would respond differently when placed on a bDMARD compared to those already pre-treated with MTX. However, whether treated with MTX or not, patients continued to have active enough disease to qualify for trial entry. Patients regardless of their MTX treatment statuses, also distributed into all three latent class response trajectories. The heterogeneity in observed responses could not be explained by MTX alone or significantly (as it did not have additional predictive effect beyond the baseline AJC). Therefore, the future focus should perhaps be on developing alternative strategies for patients with high baseline AJC already on MTX and prednisone, since we now know that this group’s response trajectory is slow and the effects small, at least in the first 16 weeks after starting a bDMARD. Waiting on response might not be a good option as we have shown that a lack of clinically significant response by 3 months is highly predictive of not attaining that by 4 months [7] Furthermore, the longer we wait, the higher the likelihood of damage to the joints. Earlier disease control has been shown to predict better long-term functional outcomes [27]. One strategy could be to use therapeutic drug monitoring information to help optimize patients for bDMARD response, as more data emerges [28].

Our study used trial data in the early phase (up to 16 weeks) following the start of bDMARD. We can only inform on early bDMARD response trajectories in polyarticular course JIA. There might be later responders but this study is unable to inform on that aspect. Also, the trial participants were recruited over a span of more than 14 years, when there had been substantial change in clinical practice, especially in earlier use of bDMARD. However, this data is still valuable for the insights possible into very early response patterns of JIA patients with varying disease severity and durations, not unlike clinical practice with heterogeneous patient populations in different practice/ environment settings (well-resourced compared to poorly-resourced). As these were trial data, we did not have a large pool of possible candidate prognostic factors to test for predictors of membership in different latent classes. We tested a limited set of covariates which are basic clinical parameters that all pediatric rheumatologists can access in daily clinical practice. Our finding of baseline AJC as predictive of post-bDMARD early response can be used by pediatric rheumatologists practicing in any setting to inform their therapeutic decisions.

## Conclusions

In this first study examining early responses to bDMARD in polyarticular course JIA patients in the first 16 weeks of trials, patients followed three possible latent class response trajectories. Baseline AJC was the strongest predictor of early response patterns. Patients with very high baseline AJC (> 30) might not respond substantively with the current approach of combination therapy including one bDMARD, and/or prednisone and/or MTX, or at least not the bDMARDs tested in this study (Etanercept, Abatacept and Tocilizumab). This group should be the target for future research for another approach to treatment. We should treat early and aggressively to prevent patients remaining in highly active states, such that they become the group that is hard to treat. Prolonging time spent in higher disease activity predispose to long-term joint damage and limitations in functional ability [27]. Future studies that closely follow JIA patients post bDMARD, but for a longer period (up to 1 year), would be helpful to clarify the question of whether it is a matter of time to respond (late responders), or that waiting really will not help most JIA patients not showing significant early response following bDMARD.

## Supplementary Information

**Additional file 1: Table A1.** Summary of Trial Designs for the 4 Included Randomized Controlled Trials (RCT). **Figure A1.** Early Latent Trajectories of Response in Juvenile Idiopathic Arthritis Patients Following Biologic DMARDs without Membership Predictor. Legend*:* Response as measured by active joint counts (AJC) that has been transformed = log (AJC + 1.8). Class 1 has high baseline AJC and slow response (19.8%). Class 2 has low baseline AJC and steady response (74.6%). Class 3 has moderate baseline AJC and a slow, steady response (5.6%). These predicted trajectories were modelled *without* membership predictors. **Table A2.** Univariable Membership Predictor Testing. * Class 1 was high baseline AJC slow response, class 2 was low baseline AJC early and sustained (plateau) response, class 3 was moderate baseline AJC and steady response. **Table A3.** Three-class latent classes probability of membership with baseline AJC as membership predictor. **Table A4.** Distribution of median AJC (25-75th percentile) for participants classified with ≥0.80 probability into the 3 latent classes (n_2_ = 450). **Figure A2.** Distribution of median active joint count (AJC) over time by latent classes of AJC, predicted by baseline AJC (n_2_ = 450). Legend: Class 1 was high baseline AJC slow response, class 2 was low baseline AJC early and sustained plateaued response, class 3 was moderate baseline AJC and progressive response.

## Data Availability

The data that support the findings of this study are available from Amgen, Bristol-Meyers Schwab, Roche and CARRA, but restrictions apply to the availability of these data, which were used under license for the current study, and so are not publicly available.

## Abbreviations

AIC: Akaike information criteria
AJC: Active joint count
ACR: American College of rheumatology
bDMARD: Biologic disease modifying antirheumatic drug
BIC: Bayesian information criteria
BMS: Bristol-Meyers Schwab
CARRA: Childhood Arthritis Rheumatology Research Alliance
DMARD: Disease modifying antirheumatic drug
ESR: Erythrocyte sedimentation rate
JIA: Juvenile idiopathic arthritis
MTX: Methotrexate
