# Supplementary material for: Latent classes of early response trajectories to biologics initiation in juvenile idiopathic arthritis: an analysis of four trials
Source: Pediatr Rheumatol Online J. 2022 Jul 30;20:57. doi: 10.1186/s12969-022-00719-1 (PMC9338501; doi:10.1186/s12969-022-00719-1)
Supplement: Supplementary file 1 — Additional file 1: Table A1. Summary of Trial Designs for the 4 Included Randomized Controlled Trials (RCT). Figure A1. Early Latent Trajectories of Response in Juvenile Idiopathic Arthritis Patients Following Biologic DMARDs without Membership Predictor. Legend: Response as measured by active joint counts (AJC) that has been transformed = log (AJC + 1.8). Class 1 has high baseline AJC and slow response (19.8%). Class 2 has low baseline AJC and steady response (74.6%). Class 3 has moderate baseline AJC and a slow, steady response (5.6%). These predicted trajectories were modelled without membership predictors. Table A2. Univariable Membership Predictor Testing. * Class 1 was high baseline AJC slow response, class 2 was low baseline AJC early and sustained (plateau) response, class 3 was moderate baseline AJC and steady response. Table A3. Three-class latent classes probability of membership with baseline AJC as membership predictor. Table A4. Distribution of median AJC (25-75th percentile) for participants classified with ≥0.80 probability into the 3 latent classes (n2 = 450). Figure A2. Distribution of median active joint count (AJC) over time by latent classes of AJC, predicted by baseline AJC (n2 = 450). Legend: Class 1 was high baseline AJC slow response, class 2 was low baseline AJC early and sustained plateaued response, class 3 was moderate baseline AJC and progressive response. [file 12969_2022_719_MOESM1_ESM.docx]

**Table A1: Summary of Trial Designs for the 4 Included Randomized Controlled Trials (RCT)**

|  | **Etanercept 2000^1^** | **TREAT** 2012^2^** | **Tocilizumab 2014^3^** | **Abatacept 2008^4^** |
| --- | --- | --- | --- | --- |
| **Study design** | Randomized withdrawal trial | Dual-arm RCT | Randomized withdrawal trial | Randomized withdrawal trial |
| **Study population** | Polyarticular course JIA | | | |
|  | -- | <12 months from diagnosis | >6 months from diagnosis | -- |
| **Biologic tested** | Etanercept | Etanercept | Tocilizumab | Abatacept |
| **Prior DMARDs allowed** | Yes but must stop | MTX < 6 weeks or none | MTX only at 10-20 mg/m^2^/wk | Only MTX allowed, stable ≥4 weeks |
| **Prior corticosteroids allowed** | Yes | Prednisone <4 weeks, stopped≥1 week | Yes, ≤0.2 mg/kg/day or ≤10 mg/day | Yes, ≤0.2 mg/kg/day or ≤10 mg/day ≥4 weeks |
| **Prior biologics** | NA | No | Yes with washout | Yes with washout |
| **In-trial DMARD allowed** | No | MTX* | Yes | Yes |
| **In-trial corticosteroids allowed** | <0.2 mg/kg/day or <10 mg/day | Yes* | Yes | Yes |
| **In-trial joint injections allowed** | No | ≤2 within 14 days from day 0 | Not specified | No, from –4 weeks before day 0 |
| **Interventions** | Etanercept 12 weeks | Randomized into Etanercept vs placebo from start | Tocilizumab 16 weeks | Abatacept 16 weeks |

Above data pertained to the first 4 months of the trial. *MTX and prednisone were part of trial interventions for TREAT. ** Only data from participants in the etanercept arm of the TREAT trial used in this analysis.

**Figure A1: Early Latent Trajectories of Response in Juvenile Idiopathic Arthritis Patients Following Biologic DMARDs without Membership Predictor**


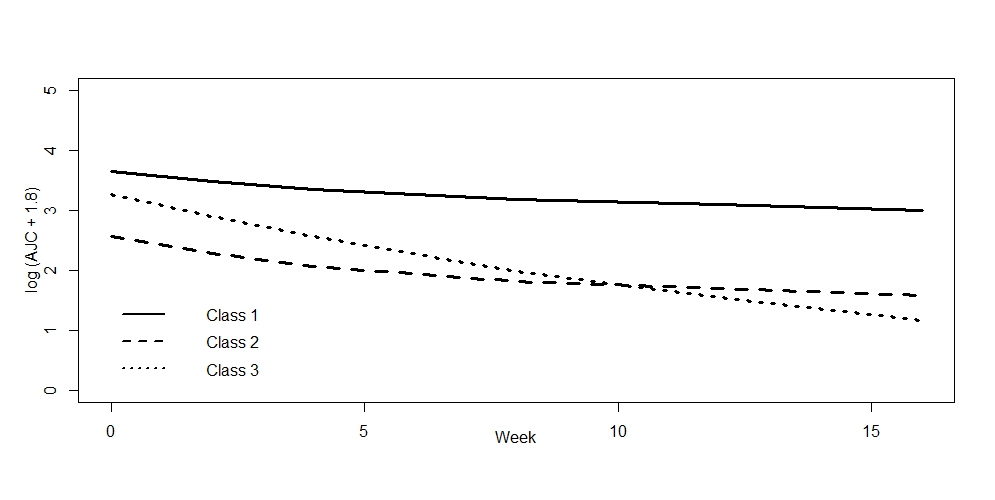


**Legend***:* Response as measured by active joint counts (AJC) that has been transformed = log (AJC +1.8). Class 1 has high baseline AJC and slow response (19.8%). Class 2 has low baseline AJC and steady response (74.6%). Class 3 has moderate baseline AJC and a slow, steady response (5.6%). These predicted trajectories were modelled *without* membership predictors.

**Table A2: Univariable Membership Predictor Testing**

| **# of Covariate** | **Name of Covariate** | **AIC** | **BIC** | **OR (95% CI)** | | |
| --- | --- | --- | --- | --- | --- | --- |
|  |  |  |  | **Class 1**  **(vs 3)*** | **Class 2**  **(vs 3)*** | **Class 1**  **(vs 2)*** |
| 1 | Duration of Disease | 3612.40 | 3719.47 | 1.01  (0.95–1.07) | 1.00  (0.91–1.10) | 1.01  (0.91–1.12) |
|  | Baseline ESR | 3777.91 | 3886.37 | 1.12  (1.02–1.23) | 1.11  (1.02–1.21) | 1.01  (1.00–1.01) |
|  | Baseline RF | 3805.74 | 3914.2 | 0.95  (0.50–1.81) | 0.87  (0.21–3.65) | 1.09  (0.25–4.87) |
|  | **Baseline AJC** | **3193.91** | **3302.38** | **1.92**  **(1.26**–**2.93)** | **0.07**  **(0.00**–**0.92)** | **29.05**  **(2.02–418.46)** |
|  | Baseline MTX | 3775.04 | 3883.51 | 0.22  (0.12–0.41) | 3.37  (0.21–54.68) | 0.07  (0.00–1.04) |
|  | Baseline Prednisone | 3805.54 | 3914 | 1.00  (0.70–1.42) | 1.32  (0.45–3.86) | 0.75  (0.25–2.25) |

***** Class 1 was high baseline AJC slow response, class 2 was low baseline AJC early and sustained (plateau) response, class 3 was moderate baseline AJC and steady response.

**Table A3: Three-class latent classes probability of membership with baseline AJC as membership predictor**

| **Class** | **Number (%)** | **Mean**  **(min-max) class probability** | **% ≥ 0.80 probability of membership (number)** | **% ≥ 0.70 probability of membership (number)** |
| --- | --- | --- | --- | --- |
| **1** | 127 (26.5) | 0.97  (0.57– 1.00) | 94.5 (120) | 96.1 (122) |
| **2** | 142 (29.7) | 0.97  (0.52– 1.00) | 93.7 (133) | 95.8 (136) |
| **3** | 210 (43.8) | 0.96  (0.57– 1.00) | 93.8 (197) | 95.2 (200) |

**Table A4:** **Distribution of median AJC (25-75^th^ percentile) for participants classified with ≥0.80 probability into the 3 latent classes (n_2_=450)**

|  | **Week 4** | **Week 8** | **Week 12** | **Week 16** |
| --- | --- | --- | --- | --- |
| **Class 1**  **High pre-treatment AJC slow response** | 27.00  (18.00, 35.00) | 21.50  (12.00, 32.25) | 18.00  (9.00, 28.00) | 12.50  (6.00, 23.00) |
| **Class 2**  **Low pre-treatment AJC early & sustained response** | 3.00  (2.00, 5.00) | 2.00  (1.00, 4.00) | 2.00  (0.00, 3.00) | 1.00  (0.00, 3.00) |
| **Class 3**  **Moderate pre-treatment AJC early & progressive response** | 10.00  (5.00, 14.00) | 7.00  (3.00, 12.00) | 5.00  (2.00, 10.00) | 3.00  (1.00, 7.00) |

**Figure A2:** **Distribution of median active joint count (AJC) over time by latent classes of AJC, predicted by baseline AJC (n_2_=450)**


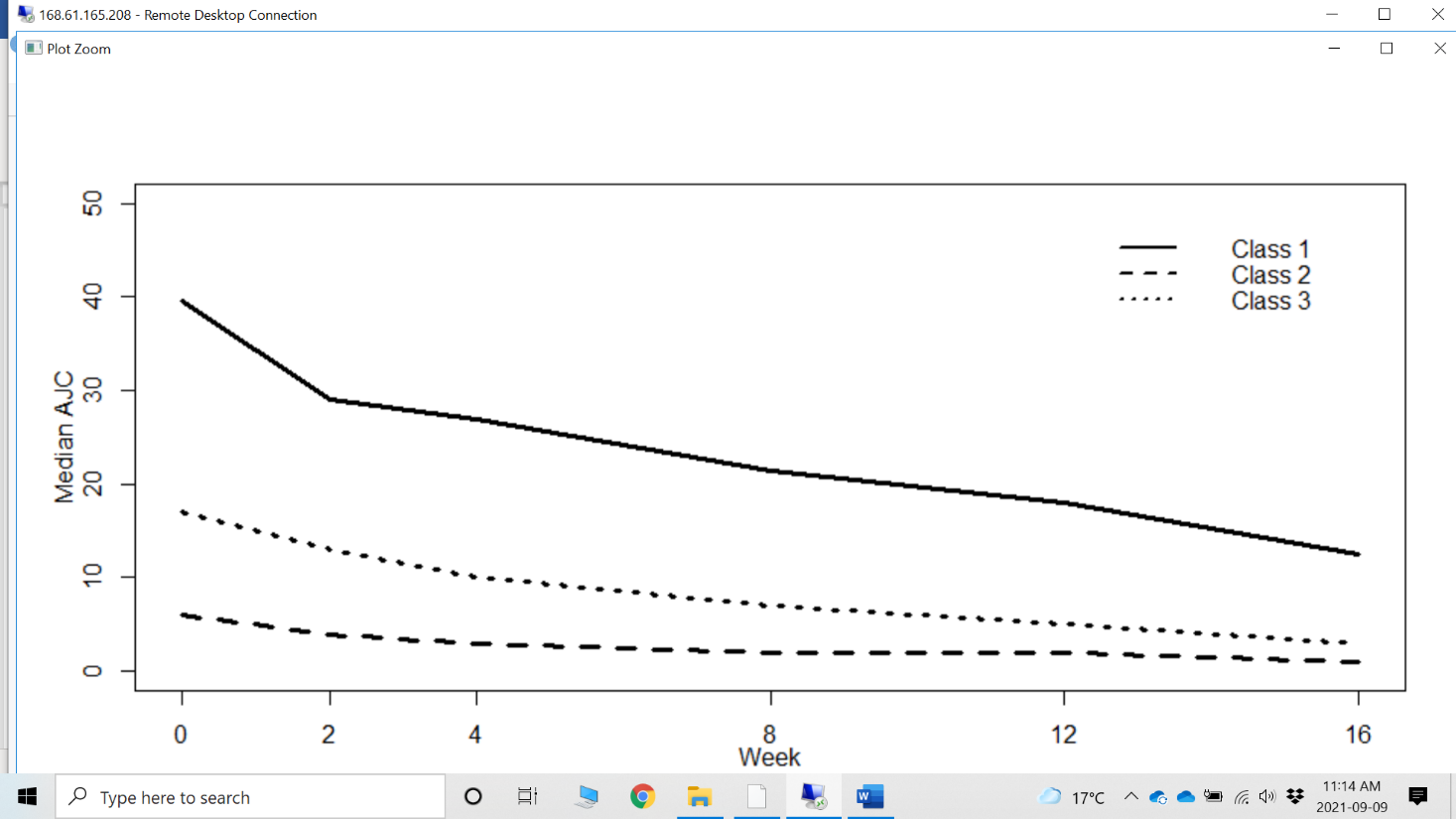


**Legend:** Class 1 was high baseline AJC slow response, class 2 was low baseline AJC early and sustained plateaued response, class 3 was moderate baseline AJC and progressive response.

**References**

1. Lovell DJ, Giannini EH, Reiff A, et al. Etanercept in children with polyarticular juvenile rheumatoid arthritis. Pediatric Rheumatology Collaborative Study Group. *N Engl J Med* 2000;342:763-9.

2. Wallace CA, Giannini EH, Spalding SJ, et al. Trial of early aggressive therapy in polyarticular juvenile idiopathic arthritis. *Arthritis Rheum* 2012;64:2012-21.

3. Brunner HI, Ruperto N, Tzaribachev Nea. A Multi-Center, Double-Blind, Randomized Withdrawal Trial of Subcutaneous Golimumab in Pediatric Patients with Active Polyarticular Course Juvenile Idiopathic Arthritis Despite Methotrexate Therapy ACR 2014 Meeting Abstracts

Number 933, 2014.

4. Ruperto N, Lovell DJ, Quartier P, et al. Abatacept in children with juvenile idiopathic arthritis: a randomised, double-blind, placebo-controlled withdrawal trial. *Lancet* 2008;372:383-91.
